# Species Diversity, Molecular Phylogeny, and Ecological Habits of *Fomitopsis* (Polyporales, Basidiomycota)

**DOI:** 10.3389/fmicb.2022.859411

**Published:** 2022-04-05

**Authors:** Shun Liu, Chang-Ge Song, Tai-Min Xu, Xing Ji, Dong-Mei Wu, Bao-Kai Cui

**Affiliations:** ^1^School of Ecology and Nature Conservation, Institute of Microbiology, Beijing Forestry University, Beijing, China; ^2^Xinjiang Production and Construction Group Key Laboratory of Crop Germplasm Enhancement and Gene Resources Utilization, Biotechnology Research Institute, Xinjiang Academy of Agricultural and Reclamation Sciences, Shihezi, China

**Keywords:** brown-rot fungi, distribution areas, multi-gene phylogeny, new species, polypore

## Abstract

*Fomitopsis* is a worldwide brown-rot fungal genus of Polyporales, which grows on different gymnosperm and angiosperm trees and has important ecological functions and economic values. In this study, species diversity, phylogenetic relationships, and ecological habits of *Fomitopsis* were investigated. A total of 195 specimens from 24 countries representing 29 species of *Fomitopsis* were studied. Based on the morphological characters and phylogenetic evidence of DNA sequences including the internal transcribed spacer (ITS) regions, the large subunit of nuclear ribosomal RNA gene (nLSU), the small subunit of nuclear ribosomal RNA gene (nSSU), the small subunit of mitochondrial rRNA gene (mtSSU), the translation elongation factor 1-α gene (TEF), and the second subunit of RNA polymerase II (RPB2), 30 species are accepted in *Fomitopsis*, including four new species: *F. resupinata*, *F. srilankensis*, *F. submeliae* and *F. yimengensis*. Illustrated descriptions of the novel species and the geographical locations of the *Fomitopsis* species are provided.

## Introduction

*Fomitopsis* P. Karst. was established by Karsten (1881) and typified by *F. pinicola* (Sw.) P. Karst. It is the type genus of Fomitopsidaceae Jülich. Species in *Fomitopsis* causes a brown rot and plays an important role in degradation and reduction of forest ecosystems (Wei and Dai, 2004). Some species of *Fomitopsis* are forest pathogens, such as, *F. nivosa* (Berk.) Gilb. & Ryvarden and *F. pinicola* (Dai, 2012); and some species are medicinal fungi, such as, *F. betulina* (Bull.) B.K. Cui, M.L. Han & Y.C. Dai has the function of antibacteria, antitumor, and antioxidant (Dai et al., 2009; Zhao et al., 2014); *F. pinicola* has the function of dispelling wind-evil and dampness, and has antitumor, antifungal, antioxidant, immunomodulation, and neuroprotective activities (Dai et al., 2009; Guler et al., 2009; Bao et al., 2015; Sun et al., 2016; Guo and Wolf, 2018).

*Fomitopsis* is a widely distributed brown-rot fungal genus and many studies have been focused on this genus since its establishment. Previously, some new species of *Fomitopsis* were described only based on morphological characteristics (Bondartsev and Singer, 1941; Cunningham, 1950; Sasaki, 1954; Ito, 1955; Reid, 1963; Ryvarden, 1972, 1984, 1988; Gilbertson and Ryvarden, 1985; Buchanan and Ryvarden, 1988; Corner, 1989; Zhao and Zhang, 1991; Reng and Zhang, 1992; Masuka and Ryvarden, 1993; Ryvarden and Gilbertson, 1993; Roy and De, 1996; Hattori, 2003; Aime et al., 2007; Stokland and Ryvarden, 2008). According to the 10th edition of the Dictionary of Fungi (Kirk et al., 2008), 32 species are accepted in *Fomitopsis* and a considerable number of these species lack molecular data.

With the progress of molecular biology technology, DNA sequencing and phylogenetic techniques have been used in the systematic study of *Fomitopsis*. Some phylogenetic studies showed that *Fomitopsis* clustered with other brown-rot fungal genera and embedded in the antrodia clade (Hibbett and Donoghue, 2001; Hibbett and Thorn, 2001; Binder et al., 2005). Subsequently, phylogenetic analyses indicated that *Fomitopsis* is polyphyletic and the taxonomic position of *Fomitopsis* is still problematic (Kim et al., 2005, 2007; Justo and Hibbett, 2011; Ortiz-Santana et al., 2013). Recently, taxonomic and phylogenetic studies on *Fomitopsis* have been carried out and several new species have been described (Li et al., 2013; Han et al., 2014, 2016; Han and Cui, 2015; Soares et al., 2017; Haight et al., 2019; Liu et al., 2019, 2021a; Zhou et al., 2021). Han et al. (2016) investigated phylogenetic relationships of *Fomitopsis* and its related genera and reported that species previously placed in *Fomitopsis* were divided into seven lineages: *Fomitopsis* s. s., *Fragifomes* B.K. Cui, M.L. Han & Y.C. Dai, *Niveoporofomes* B.K. Cui, M.L. Han & Y.C. Dai, *Rhodofomes* Kotl. & Pouzar, *Rhodofomitopsis* B.K. Cui, M.L. Han & Y.C. Dai, *Rubellofomes* B.K. Cui, M.L. Han & Y.C. Dai, and *Ungulidaedalea* B.K. Cui, M.L. Han & Y.C. Dai.

To date, 127 taxa of *Fomitopsis* have been recorded in the database of Index Fungorum and 138 taxa of *Fomitopsis* have been recorded in the database of MycoBank, however, it includes a large number of synonymous taxa and invalid published names. In the current study, phylogenetic analysis of *Fomitopsis* was carried out based on the combined sequence dataset of ITS + nLSU + mtSSU + nSSU + RPB2 + TEF rRNA and/or rDNA gene regions. Combining with morphological characters and molecular evidence, four new species, *F. resupinata*, *F. srilankensis*, *F. submeliae*, and *F. yimengensis* have been discovered.

## Materials and Methods

### Morphological Studies

The examined specimens were deposited at the herbarium of the Institute of Microbiology, Beijing Forestry University (BJFC), and some duplicates were deposited at the Institute of Applied Ecology, Chinese Academy of Sciences, China (IFP). Morphological descriptions and abbreviations used in this study followed Cui et al. (2019) and Shen et al. (2019).

### DNA Extraction and Sequencing

The procedures for DNA extraction and polymerase chain reaction (PCR) used in this study were the same as described by Han et al. (2016) and Liu et al. (2019, 2022). The ITS regions were amplified with the primer pairs ITS4 and ITS5, the nLSU regions were amplified with the primer pairs LR0R and LR7, the nSSU regions were amplified with the primer pairs NS1 and NS4, the mtSSU regions were amplified with the primer pairs MS1 and MS2, the RPB2 gene was amplified with the primer pairs fRPB2-f5F and bRPB2-7.1R, and the TEF gene was amplified with the primer pairs EF1-983F and EF1-1567R (White et al., 1990; Matheny, 2005; Rehner and Buckley, 2005).

The PCR cycling schedules for different DNA sequences of ITS, nLSU, nSSU, mtSSU, RPB2, and TEF genes used in this study followed those used in Liu et al. (2019); Shen et al. (2019), Zhu et al. (2019), and Ji et al. (2022) with some modifications. The PCR products were purified and sequenced at Beijing Genomics Institute, China, with the same primers. All newly generated sequences were submitted to GenBank and are listed in Table 1.

**TABLE 1:** A list of species, specimens, and GenBank accession number of sequences used for phylogenetic analyses in this study.

Species name	Sample no.	Locality	GenBank accessions					
			
			ITS	nLSU	mtSSU	nSSU	RPB2	TEF
*Antrodia heteromorpha*	Dai 12755	United States	KP715306	KP715322	KR606009	KR605908	KR610828	KP715336
*Antrodia serpens*	Dai 14850	Poland	MG787582	MG787624	MG787674	MG787731	MG787798	MG787849
*Antrodia subserpens*	Cui 8310	China	KP715310	KP715326	MG787677	MG787732	KT895888	KP715340
*Antrodia tanakae*	Cui 9743	China	KR605814	KR605753	KR606014	KR605914	KR610833	KR610743
*Brunneoporus cyclopis*	Miettinen 9166.1	Indonesia	KU866249	MG787627	MG787679	MG787737	MG787802	KU866242
*Brunneoporus kuzyana*	JV 0909/37	Czech Republic	KU866267	MG787628	MG787680	MG787738	MG787803	KU866221
*Brunneoporus malicolus*	Cui 7258	China	MG787586	MG787631	MG787683	MG787741	MG787806	MG787853
*Buglossoporus eucalypticola*	Dai 13660	China	KR605808	KR605747	KR606007	KR605906	KR610825	KR610736
*Brunneoporus quercinus*	JV 0906/15-J	United States	KR605800	KR605739	KR606001	KR605898	KR610819	KR610729
*Daedalea circularis*	Cui 10125	China	JQ780411	KP171220	KR605978	KR605875	KR610799	KR610708
*Daedalea modesta*	Cui 10124	China	KR605791	KR605730	KR605985	KR605882	KR610805	KR610715
*Daedalea quercina*	Dai 12659	Finland	KP171208	KP171230	KR605990	KR605887	KR610810	KR610719
*Daedalea radiata*	Cui 8575	China	KP171210	KP171233	KR605991	KR605888	KR610811	KR610720
*Flavidoporia mellita*	VS 3315	Russia	KC543140	KC543140	–	–	–	–
*Flavidoporia pulverulenta*	LY BR 3450	France	JQ700280	JQ700280	–	–	–	–
*Flavidoporia pulvinascens*	X 1372	Finland	JQ700286	JQ700286	–	–	–	–
*Fomitopsis abieticola*	Cui 10521	China	MN148231	OL621245 *	OL621756 *	–	–	MN161746
*Fomitopsis abieticola*	Cui 10532 holotype	China	MN148230	OL621246 *	OL621757 *	–	MN158174	MN161745
*Fomitopsis bambusae*	Dai 22110	China	MW937874	MW937881	MW937888	MW937867	MZ082974	MZ082980
*Fomitopsis bambusae*	Dai 22116 holotype	China	MW937876	MW937883	MW937890	MW937869	–	–
*Fomitopsis betulina*	Cui 17121	China	OL621853 *	OL621242 *	OL621753 *	OL621779 *	OL588969 *	OL588982 *
*Fomitopsis betulina*	Cui 10756	China	KR605797	KR605736	KR605997	KR605894	KR610815	KR610725
*Fomitopsis betulina*	Dai 11449	China	KR605798	KR605737	KR605998	KR605895	KR610816	KR610726
*Fomitopsis bondartsevae*	X 1207	China	JQ700277	JQ700277	–	–	–	–
*Fomitopsis bondartsevae*	X 1059	China	JQ700275	JQ700275	–	–	–	–
*Fomitopsis cana*	Cui 6239	China	JX435777	JX435775	KR605934	KR605826	KR610761	KR610661
*Fomitopsis cana*	Dai 9611 holotype	China	JX435776	JX435774	KR605933	KR605825	KR610762	KR610660
*Fomitopsis caribensis*	Cui 16871 holotype	Puerto Rico	MK852559	MK860108	MK860116	MK860124	MK900474	MK900482
*Fomitopsis durescens*	Overholts 4215	United States	KF937293	KF937295	KR605941	KR605835	–	–
*Fomitopsis durescens*	O 10796	Venezuela	KF937292	KF937294	KR605940	KR605834	KR610766	KR610669
*Fomitopsis eucalypticola*	Cui 16594	Australia	MK852560	MK860110	MK860118	MK860126	MK900476	MK900483
*Fomitopsis eucalypticola*	Cui 16598 holotype	Australia	MK852562	MK860113	MK860121	MK860129	MK900479	MK900484
*Fomitopsis ginkgonis*	Cui 17170 holotype	China	MK852563	MK860114	MK860122	MK860130	MK900480	MK900485
*Fomitopsis ginkgonis*	Cui 17171	China	MK852564	MK860115	MK860123	MK860131	MK900481	MK900486
*Fomitopsis hemitephra*	O 10808	Australia	KR605770	KR605709	KR605947	KR605841	–	KR610675
*Fomitopsis hengduanensis*	Cui 16259 holotype	China	MN148232	OL621247 *	OL621758 *	OL621782 *	MN158175	MN161747
*Fomitopsis hengduanensis*	Cui 17056	China	MN148233	OL621248 *	OL621759 *	OL621783 *	MN158176	MN161748
*Fomitopsis iberica*	Dai 6614	China	MG787591	MG787637	MG787689	MG787747	MG787812	MG787858
*Fomitopsis iberica*	O 10811	Italy	KR605772	KR605711	–	KR605843	KR610772	KR610677
*Fomitopsis kesiyae*	Cui 16437 holotype	Vietnam	MN148234	OL621249 *	OL621760 *	OL621784 *	MN158177	MN161749
*Fomitopsis kesiyae*	Cui 16466	Vietnam	MN148235	OL621250 *	OL621761 *	OL621785 *	MN158178	MN161750
*Fomitopsis massoniana*	Cui 11304 holotype	China	MN148239	OL621251 *	OL621762 *	–	–	MN161754
*Fomitopsis massoniana*	Cui 11288	China	MN148238	OL621252 *	OL621763 *	–	MN158179	MN161753
*Fomitopsis meliae*	Roberts GA863	United Kingdom	KR605775	KR605714	KR605953	KR605848	–	KR610682
*Fomitopsis meliae*	Ryvarden 16893	Unknown	KR605776	KR605715	KR605954	KR605849	KR610775	KR610681
*Fomitopsis mounceae*	DR-366	United States	KF169624	–	–	–	KF169693	KF178349
*Fomitopsis mounceae*	JAG-08-19	United States	KF169626	–	–	–	KF169695	KF178351
*Fomitopsis nivosa*	Man 09	Brazil	MF589766	MF590166	–	–	–	–
*Fomitopsis nivosa*	JV 0509/52-X	China	KR605779	KR60571	KR605957	KR605853	KR610777	KR610686
*Fomitopsis ochracea*	ss 5	Canada	KF169609	–	–	–	KF169678	KF178334
*Fomitopsis ochracea*	ss 7	Canada	KF169610	–	–	–	KF169679	KF178335
*Fomitopsis ostreiformis*	Cui 18217	Malaysia	OL621855	OL621244 *	OL621755 *	OL621781 *	OL588970 *	OL588984 *
*Fomitopsis ostreiformis*	IRET 22	Gabon	KY449363	–	–	–	–	–
*Fomitopsis ostreiformis*	LDCMY 21	India	KY111252	–	–	–	–	–
*Fomitopsis palustris*	Cui 7597	China	KP171213	KP171236	KR605958	KR605854	KR610778	KR610687
*Fomitopsis palustris*	Cui 7615	China	KR605780	KR605719	KR605959	KR605855	KR610779	KR610688
*Fomitopsis pinicola*	LT 319	Estonia	KF169652	–	–	–	KF169721	KF178377
*Fomitopsis pinicola*	AT Fp 1	Sweden	MK208852	–	–	–	MK236362	MK236359
***Fomitopsis resupinata***	Cui 6697	**China**	OL621842 *	OL621231 *	OL621745 *	OL621768 *	OL588960 *	OL588971 *
***Fomitopsis resupinata***	Dai 10819 holotype	**China**	OL621843 *	OL621232 *	OL621746 *	OL621769 *	OL588961 *	OL588972 *
*Fomitopsis roseoalba*	AS 1496	Brazil	KT189139	KT189141	–	–	–	–
*Fomitopsis roseoalba*	AS 1566	Brazil	KT189140	KT189142	–	–	–	–
*Fomitopsis schrenkii*	JEH-144	United States	KF169621	–	–	–	MK208857	MK236355
*Fomitopsis schrenkii*	JEH-150 holotype	United States	KF169622	–	–	–	MK208858	MK236356
***Fomitopsis srilankensis***	Dai 19528 holotype	**Sri Lanka**	OL621844 *	OL621233 *	OL621747 *	OL621770 *	OL588962 *	OL588973 *
***Fomitopsis srilankensis***	Dai 19539	**Sri Lanka**	OL621845 *	OL621234 *	OL621748 *	OL621771 *	OL588963 *	OL588974 *
***Fomitopsis submeliae***	Dai 10035	**China**	KR605774	KR605713	KR605952	KR605847	–	KR610683
***Fomitopsis submeliae***	Dai 18324	**Vietnam**	OL621846 *	OL621235 *	OL621749 *	OL621772 *	–	OL588975 *
***Fomitopsis submeliae***	Dai 9719	**China**	OL621847 *	OL621236 *	OL621750 *	OL621773 *	–	OL588976 *
***Fomitopsis submeliae***	Dai 18559 holotype	**Malaysia**	OL621848 *	OL621237 *	OL621751 *	OL621774 *	OL588964 *	OL588977 *
***Fomitopsis submeliae***	Cui 6305	**China**	OL621849 *	OL621238 *	OL621752 *	OL621775 *	OL588965 *	OL588978 *
*Fomitopsis subpinicola*	Cui 9836 holotype	China	MN148249	OL621253 *	OL621764 *	–	MN158181	MN161764
*Fomitopsis subpinicola*	Dai 11206	China	MN148252	OL621254 *	OL621765 *	–	MN158183	MN161767
*Fomitopsis subtropica*	Dai 18566	China	OL621854 *	OL621243 *	OL621754 *	OL621780 *	–	OL588983 *
*Fomitopsis subtropica*	Cui 10578 holotype	China	KR605787	KR605726	KR605971	KR605867	KR610791	KR610698
*Fomitopsis subtropica*	Cui 10140	China	JQ067651	JX435771	KR605969	KR605865	KR610789	KR610699
*Fomitopsis tianshanensis*	Cui 16821 holotype	China	MN148258	OL621255 *	OL621766 *	OL621786 *	–	MN161773
*Fomitopsis tianshanensis*	Cui 16823	China	MN148259	OL621256 *	OL621767 *	OL621787 *	–	MN161774
***Fomitopsis yimengensis***	Cui 5027 holotype	**China**	OL621850 *	OL621239 *	OL621839 *	OL621776 *	OL588966 *	OL588979 *
***Fomitopsis yimengensis***	Cui 5031	**China**	OL621851 *	OL621240 *	OL621840 *	OL621777 *	OL588967 *	OL588980 *
***Fomitopsis yimengensis***	Cui 5111	**China**	OL621852 *	OL621241 *	OL621841 *	OL621778 *	OL588968 *	OL588981 *
*Laetiporus sulphureus*	Cui 12388	China	KR187105	KX354486	KX354560	KX354518	KX354652	KX354607
*Laetiporus zonatus*	Cui 10404	China	KF951283	KF951308	KX354593	KX354551	KT894797	KX354639
*Neoantrodia primaeva*	Dai 11156	China	MG787598	MG787645	MG787699	MG787761	MG787820	–
*Neoantrodia serialis*	JV 1509/5	Czech Republic	KT995120	KT995143	–	–	–	KU052726
*Neoantrodia serrate*	Dai 7626	China	KR605812	KR605751	KR606012	KR605912	KR610831	KR610740
*Neoantrodia subserialis*	Cui 9706	China	KR605811	KR605750	KR606010	KR605910	KR610829	KR610741
*Niveoporofomes spraguei*	4638	France	KR605784	KR605723	KR605966	KR605862	KR610786	KR610696
*Niveoporofom spraguei*	JV 0509/62	United States	KR605786	KR605725	KR605968	KR605864	KR610788	KR610697
*Rhodofomes cajanderi*	Cui 9888	China	KC507156	KC507166	KR605936	KR605828	KR610764	KR610662
*Rhodofomes incarnates*	Cui 10348	China	KC844848	KC844853	KR605949	KR605844	KR610773	KR610679
*Rhodofomes rosea*	Cui 10520	China	KC507162	KC507172	KR605963	KR605859	KR610783	KR610692
*Rhodofomes subfeei*	Dai 11887	China	KC507160	KC507170	KR605973	KR605870	KR610794	KR610703
*Rhodofomitopsis feei*	Ryvarden 37603	Venezuela	KC844850	KC844855	KR605944	KR605838	KR610768	KR610670
*Rhodofomitopsis lilacinogilva*	Schigel 5193	Australia	KR605773	KR605712	KR605945	KR605846	KR610774	KR610680
*Rhodofomitopsis monomitic*	Dai 16894	China	KY421733	KY421735	MG787711	MG787781	MG787826	MG787869
*Rubellofomes cystidiatus*	Cui 5481	China	KF937288	KF937291	KR605938	KR605832	KR610765	KR610667
*Rubellofomes cystidiatus*	Yuan 6304	China	KR605769	KR605708	KR605939	KR605833	–	KR610668
*Rubellofomes minutisporus*	Rajchenberg 10661	Argentina	KR605777	KR605716	–	KR605850	–	–
*Subantrodia juniperina*	03010/1a	United States	MG787606	MG787653	MG787712	MG787782	MG787831	MG787873
*Subantrodia uzbekistanica*	Dai 17104	Uzbekistan	KX958182	KX958186	–	–	–	–
*Subantrodia uzbekistanica*	Dai 17105	Uzbekistan	KX958183	KX958187	–	–	–	–
*Ungulidaedalea fragilis*	Cui 10919	China	KF937286	KF937290	KR605946	KR605840	KR610770	KR610674

*Newly generated sequences for this study. New species are shown in bold.

### Phylogenetic Analyses

Sequences were aligned with additional sequences downloaded from GenBank (Table 1) using BioEdit (Hall, 1999) and ClustalX (Thompson et al., 1997). Alignment was manually adjusted to allow maximum alignment and to minimize gaps. Sequence alignment was deposited at TreeBase (submission ID 29193).^1^ The sequences of *Laetiporus sulphureus* (Bull.) Murrill and *L. zonatus* B.K. Cui & J. Song, obtained from GenBank, were used as outgroups for the phylogenetic analyses of *Fomitopsis*.

Phylogenetic analyses approaches used in this study followed Sun et al. (2020) and Liu et al. (2021b). The congruences of the 6-genes (ITS, nLSU, nSSU, mtSSU, RPB2, and TEF) were evaluated with the incongruence length difference (ILD) test (Farris et al., 1994) implemented in PAUP* 4.0b10 (Swofford, 2002), under heuristic search and 1,000 homogeneity replicates. Maximum parsimony (MP) analysis was performed in PAUP* version 4.0b10 (Swofford, 2002). Clade robustness was assessed using a bootstrap (BT) analysis with 1,000 replicates (Felsenstein, 1985). Descriptive tree statistics tree length (TL), consistency index (CI), retention index (RI), rescaled consistency index (RC), and homoplasy index (HI) were calculated for each Most Parsimonious Tree (MPT) generated. Maximum Likelihood (ML) analysis was performed in RAxmL v.7.2.8 with a GTR + G + I model (Stamatakis, 2006). Bayesian inference (BI) was calculated by MrBayes 3.1.2 (Ronquist and Huelsenbeck, 2003) with a general time reversible (GTR) model of DNA substitution and a gamma distribution rate variation across sites determined by MrModeltest 2.3 (Posada and Crandall, 1998; Nylander, 2004). The branch support was evaluated with a bootstrapping method of 1,000 replicates (Hillis and Bull, 1993).

Branches that received bootstrap supports for MP, ML greater than or equal to 75%, and Bayesian posterior probabilities (BPP) greater than or equal to 0.95 were considered as significantly supported. The phylogenetic tree was visualized using FigTree v1.4.2.^2^

## Results

### Molecular Phylogeny

**FIGURE 1:**
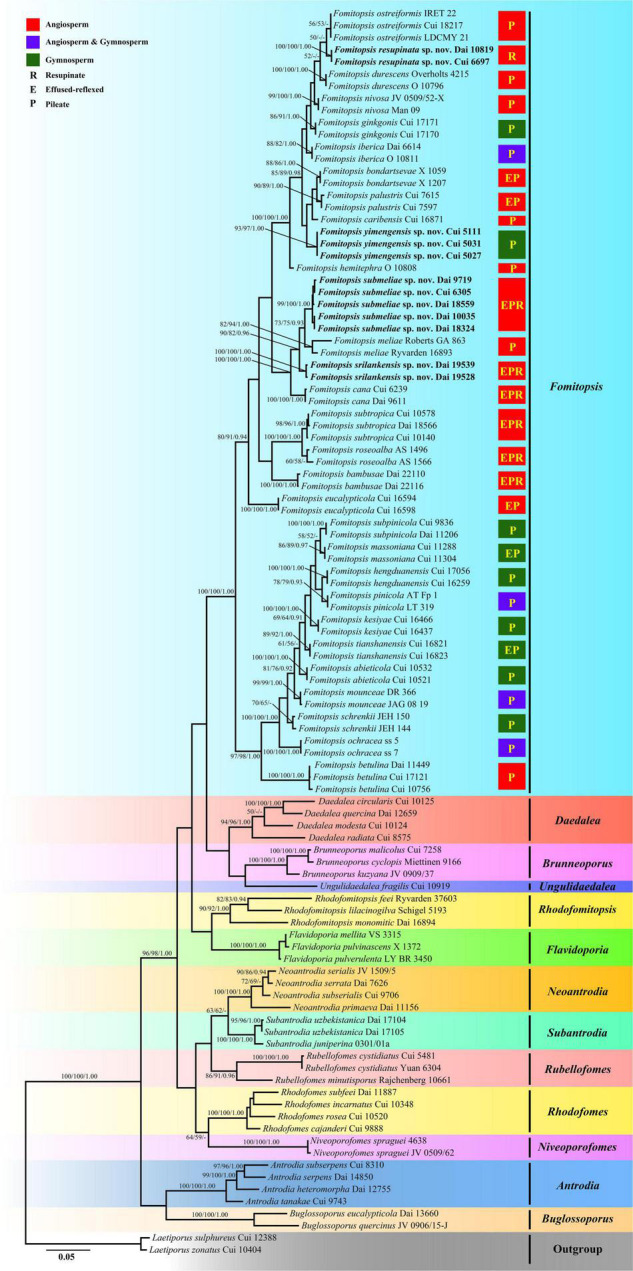
Maximum likelihood tree illustrating the phylogeny of *Fomitopsis* and its related genera in the antrodia clade based on the combined sequences dataset of ITS + nLSU + nSSU + mtSSU + RPB2 + TEF. Branches are labeled with maximum likelihood bootstrap higher than 50%, parsimony bootstrap proportions higher than 50% and Bayesian posterior probabilities more than 0.90, respectively. Bold names = New species.

The combined 6-gene sequences dataset for phylogenetic analyses had an aligned length of 4,626 characters including gaps (610 characters for ITS, 1,346 characters for nLSU, 526 characters for mtSSU, 1,009 characters for nSSU, 648 characters for RPB2, 487 characters for TEF), of which 3,113 characters were constant, 240 were variable and parsimony-uninformative, and 1,273 were parsimony-informative. MP analysis yielded 12 equally parsimonious trees (TL = 6,756, CI = 0.366, RI = 0.722, RC = 0.264, HI = 0.634). The best model for the concatenate sequence dataset estimated and applied in the Bayesian inference was GTR + I + G with equal frequency of nucleotides, lset nst = 6 rates = invgamma; prset statefreqpr = dirichlet (1,1,1,1). Bayesian analysis resulted in a concordant topology with an average standard deviation of split frequencies = 0.008762. ML analysis resulted in a similar topology as MP and Bayesian analyses, and only the ML topology is shown in Figure 1. The phylogenetic trees inferred from ITS + nLSU + nSSU + mtSSU + RPB2 + TEF gene sequences were obtained from 103 fungal samples representing 65 taxa of *Fomitopsis* and its related genera within the antrodia clade. Also, 64 samples representing 30 taxa of *Fomitopsis* clustered together and separated from other genera.

### Taxonomy

**FIGURE 2:**
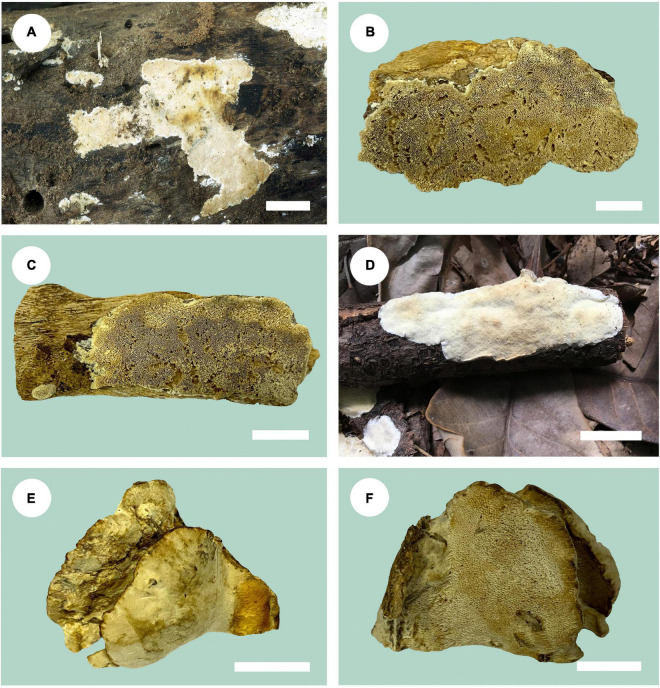
Basidiomata of *Fomitopsis* species. **(A)** *F. resupinata*; **(B,C)** *F. srilankensis*; **(D)** *F. submeliae*; **(E,F)** *F. yimengensis* (scale bars: b, f = 1.5 cm; a, c, e = 2 cm; d = 3 cm).

**FIGURE 3:**
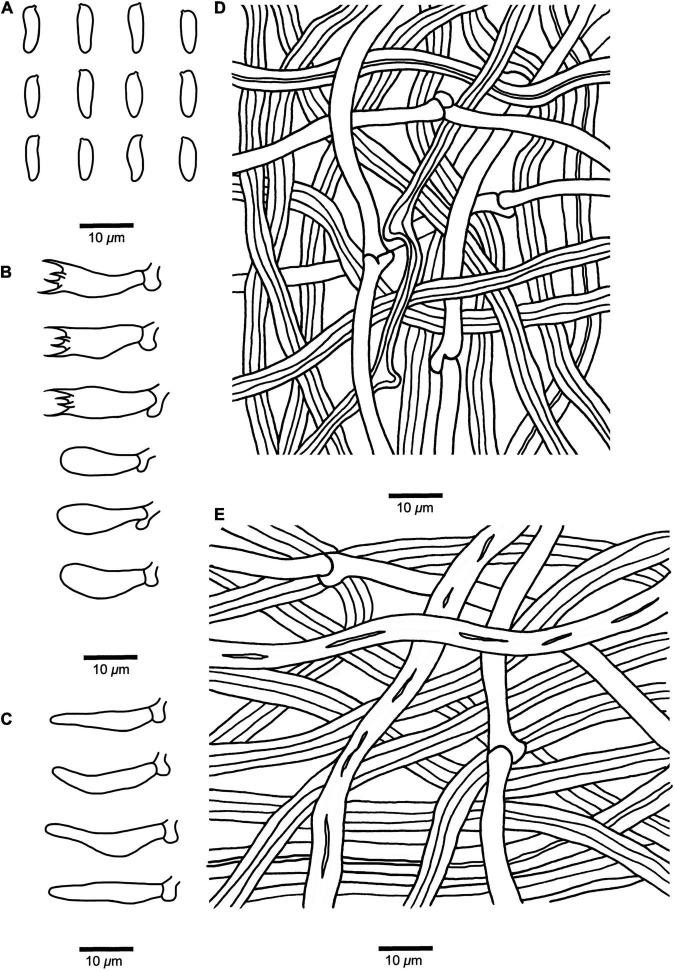
Microscopic structures of *Fomitopsis resupinata* (Holotype, *Dai 10819*). **(A)** Basidiospores. **(B)** Basidia and basidioles. **(C)** Cystidioles. **(D)** Hyphae from trama. **(E)** Hyphae from context. Drawings by: Shun Liu.

***Fomitopsis resupinata*** B.K. Cui & Shun Liu, **sp. nov.** (Figures 2A, 3).

MycoBank: MB 842873.

*Diagnosis* — ***Fomitopsis resupinata*** is characterized by its resupinate basidiomata with cream to buff pore surface when fresh, becoming pinkish buff to honey-yellow upon drying and cylindrical to slightly allantoid basidiospores (7.2–9 × 2.7–3.3 μm).

*Holotype* — **CHINA**. Hainan Province, Changjiang County, Bawangling Nature Reserve, on fallen trunk of *Mangifera infica*, 9 May 2009, *Dai 10819* (BJFC 010395).

*Etymology* — “*resupinata*” (Lat.): refers to the resupinate basidiomata.

*Fruiting body* — Basidiomata annual, resupinate, not easily separated from substrate, without odor or taste when fresh, becoming corky and light in weight upon drying; up to 9 cm long, 8.4 cm wide, and 8 mm thick at center. Pore surface cream to buff when fresh, becoming pinkish buff to honey-yellow upon drying; pores round to angular, 4–6 per mm; dissepiments slightly thick, entire. Context very thin, corky, cream to buff, up to 3 mm thick. Tubes concolorous with pore surface, corky, up to 5 mm long. Tissues unchanged in KOH.

*Hyphal structure* — Hyphal system dimitic; generative hyphae bearing clamp connections; skeletal hyphae IKI–, CB–.

*Context* — Generative hyphae infrequent, hyaline, thin-walled, rarely branched, 2–3.4 μm in diam; skeletal hyphae dominant, yellowish brown to cinnamon brown, thick-walled with a narrow lumen to subsolid, unbranched, straight, interwoven, 3.2–5.5 μm in diam.

*Tubes* — Generative hyphae infrequent, hyaline, thin-walled, rarely branched, 1.9–3 μm in diam; skeletal hyphae dominant, yellowish brown to cinnamon brown, thick-walled with a wide to narrow lumen, unbranched, more or less straight, interwoven, 2–5 μm in diam. Cystidia absent; cystidioles occasionally present, fusoid, hyaline, thin-walled, 13.2–22 × 3.2–4.3 μm. Basidia clavate, bearing four sterigmata and a basal clamp connection, 13.5–17.4 × 4.8–6.2 μm; basidioles dominant, similar to basidia but smaller.

*Spores* — Basidiospores cylindrical to slightly allantoid, hyaline, thin-walled, smooth, IKI–, CB–, (7–)7.2–9(–9.5) × (2.6–)2.7–3.3(–3.5) μm, L = 8.14 μm, W = 2.93 μm, Q = 2.46–3.52 (*n* = 60/2).

*Type of rot* — Brown rot.

*Notes* — Phylogenetically, *Fomitopsis resupinata* was closely related to *F. durescens* (Overh. ex J. Lowe) Gilb. & Ryvarden, *F. nivosa* and *F. ostreiformis* (Berk.) T. Hatt (Figure 1). They share similar sized pores, but *F. durescens* differs in its pileate basidiomata with a white to cream pore surface when fresh, ochraceous when dry, smaller and narrower cylindrical basidiospores (6–8 × 1.5–2.5 μm; Gilbertson and Ryvarden, 1986); *F. nivosa* differs by having pileate basidiomata with a cream to pale sordid brown or tan pore surface, and has a distribution in Asia, North America, and South America (Núñez and Ryvarden, 2001; Han et al., 2016); *F. ostreiformis* differs in its effused reflexed to pileate basidiomata, soft when fresh, hard when dry, a trimitic hyphal system, smaller and cylindrical basidiospores (4.2–5.6 × 1.4–2.6 μm; De, 1981). *Fomitopsis bambusae* Y.C. Dai, Meng Zhou & Yuan Yuan and *F. cana* B.K. Cui, Hai J. Li & M.L. Han also distribute in Hainan Province of China, but *F. bambusae* differs by having bluish-gray to pale mouse-gray pore surface when fresh, becoming mouse-gray to dark gray when dry, smaller pores (6–9 per mm), smaller and cylindrical to oblong ellipsoid basidiospores (4.2–6.1 × 2–2.3 μm), and grows on bamboo (Zhou et al., 2021); *F. cana* differs by having cream to straw colored pore surface when young which becoming mouse-gray to dark gray with age, a trimitic hyphal system, smaller and cylindrical to oblong-ellipsoid basidiospores (5–6.2 × 2.1–3 μm; Li et al., 2013).

Additional specimen (paratype) examined — **CHINA**. Hainan Province, Wanning County, on fallen angiosperm trunk, 14 May 2009, *Cui 6697* (BJFC 004551).

**FIGURE 4:**
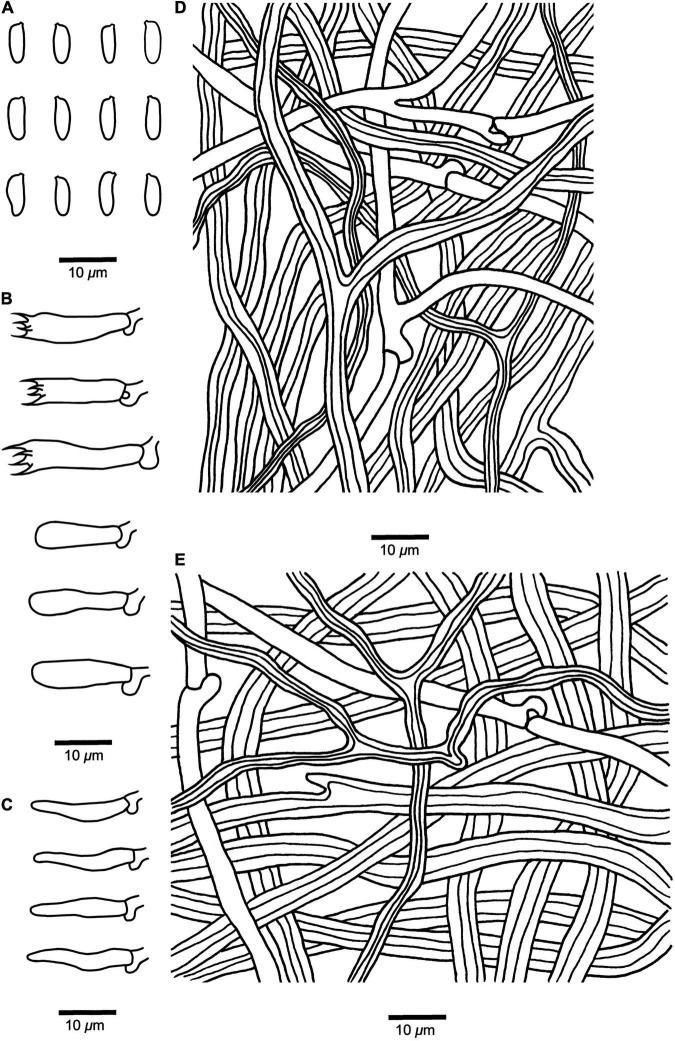
Microscopic structures of *Fomitopsis srilankensis* (Holotype, *Dai 19539*). **(A)** Basidiospores. **(B)** Basidia and basidioles. **(C)** Cystidioles. **(D)** Hyphae from trama. **(E)** Hyphae from context. Drawings by: Shun Liu.

***Fomitopsis srilankensis*** B.K. Cui & Shun Liu, **sp. nov.** (Figures 2B,C, 4).

MycoBank: MB 842874.

*Diagnosis* — ***Fomitopsis srilankensis*** is characterized by its resupinate to effused-reflexed or pileate basidiomata with pale mouse-gray to honey-yellow pileal surface when dry, buff to cinnamon-buff pore surface when dry, and cylindrical basidiospores (5.5–6.6 × 1.9–2.5 μm).

*Holotype* — **Sri Lanka**. Wadduwa, South Bolgoda Lake, on angiosperm stump, February 28, 2018, *Dai 19539* (BJFC 031218).

*Etymology* — “*srilankensis*” (Lat.): refers to the species occurrence in Sri Lanka.

*Fruiting body* — Basidiomata annual, resupinate to effused-reflexed or pileate, without odor or taste, becoming corky and light in weight upon drying. Pilei applanate, semicircular to elongated, projecting up to 2.5 cm, 1.3 cm wide, and 7 mm thick at base; resupinate part up to 8.6 cm long, 2.8 cm wide and 1.8 mm thick at center. Pileal surface pale mouse-gray to honey-yellow when dry, glabrous, sulcate, azonate; margin obtuse, concolorous with the pileal surface. Pore surface buff to cinnamon-buff when dry; pores round to angular, 5–8 per mm; dissepiments thick, entire. Context cream to pinkish buff, corky, up to 4 mm thick. Tubes concolorous with pore surface, corky, up to 3 mm long. Tissues unchanged in KOH.

*Hyphal structure* — Hyphal system dimitic; generative hyphae bearing clamp connections; skeletal hyphae IKI–, CB–.

*Context* — Generative hyphae infrequent, hyaline, thin-walled, rarely branched, 2–3.4 μm in diam; skeletal hyphae dominant, yellowish brown to cinnamon brown, thick-walled with a wide to narrow lumen, occasionally branched, more or less straight, interwoven, 2.4–5.8 μm in diam.

*Tubes* — Generative hyphae infrequent, hyaline, thin-walled, occasionally branched, 1.9–3 μm in diam; skeletal hyphae dominant, yellowish brown to cinnamon brown, thick-walled with a wide to narrow lumen, occasionally branched, more or less straight, interwoven, 2–5 μm in diam. Cystidia absent; cystidioles occasionally present, fusoid, hyaline, thin-walled, 10.5–15.5 × 2.4–3.2 μm. Basidia clavate, bearing four sterigmata and a basal clamp connection, 8.9–15.8 × 4.8–6.2 μm; basidioles dominant, similar to basidia but smaller.

*Spores* — Basidiospores cylindrical, hyaline, thin-walled, smooth, IKI–, CB–, (5.3–)5.5–6.6(–6.7) × (1.7–)1.9–2.5 μm, L = 6.11 μm, W = 2.16 μm, Q = 2.52–2.96 (*n* = 60/2).

*Type of rot* — Brown rot.

*Notes* — In the phylogenetic tree, *Fomitopsis srilankensis* grouped together with *F. cana*, *F. meliae* (Underw.) Gilb. and *F. submeliae* (Figure 1). Morphologically, they share similar sized pores, but *F. cana* differs in having pale mouse-gray to dark gray pileal surface, cream to straw colored pore surface when young and turning mouse-gray to dark gray with age, a trimitic hyphal system and wider basidiospores (5–6.2 × 2.1–3 μm; Li et al., 2013); *F. meliae* differs in having pileate basidiomata, glabrous to minutely tomentose pileal surface, ochraceous pore surface and larger basidiospores (6–8 × 2.5–3 μm; Gilbertson, 1981; Núñez and Ryvarden, 2001); *F. submeliae* differs from *F. srilankensis* by its cream pileal surface when fresh, becoming buff to buff yellow when dry, cream to pinkish buff pore surface when fresh, becoming cream to clay-buff when dry and smaller basidiospores (4–5 × 1.9–2.4 μm).

Additional specimen (paratype) examined — **Sri Lanka**. Wadduwa, South Bolgoda Lake, on fallen angiosperm trunk, February 28, 2018, *Dai 19528* (BJFC 031207).

**FIGURE 5:**
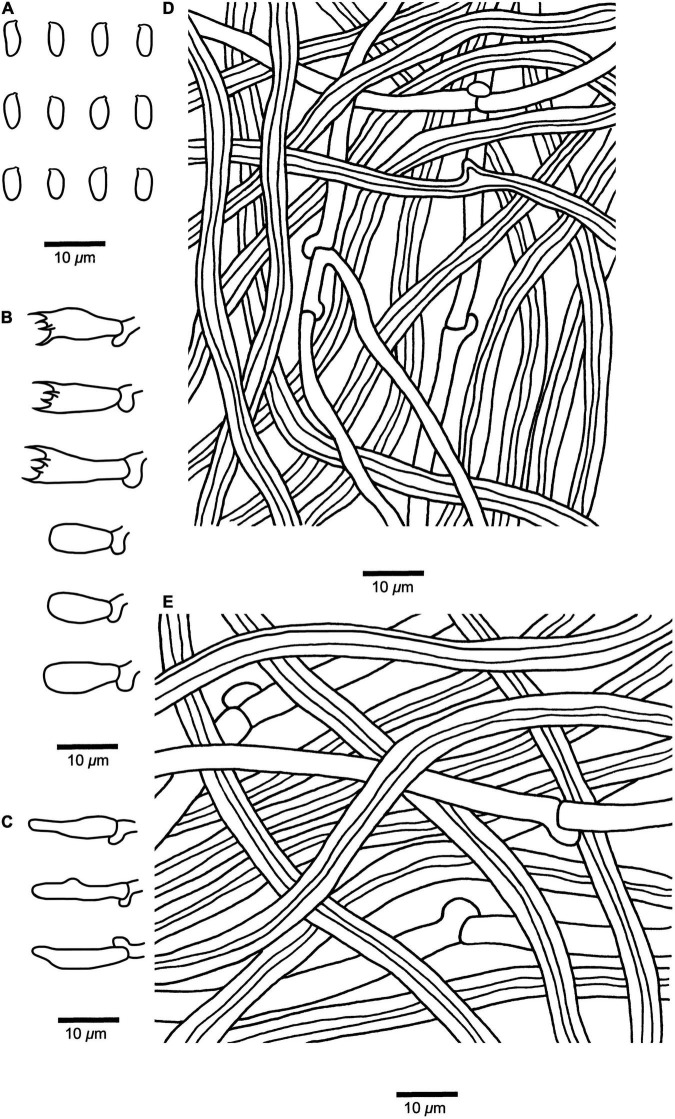
Microscopic structures of *Fomitopsis submeliae* (Holotype, *Dai 18559*). **(A)** Basidiospores. **(B)** Basidia and basidioles. **(C)** Cystidioles. **(D)** Hyphae from trama. **(E)** Hyphae from context. Drawings by: Shun Liu.

***Fomitopsis submeliae*** B.K. Cui & Shun Liu, **sp. nov.** (Figures 2D, 5).

MycoBank: MB 842875.

*Diagnosis* — ***Fomitopsis submeliae*** is characterized by its effused-reflexed basidiomata with several small imbricate pilei protruding from a large resupinate part, pale mouse-gray to grayish brown pileal surface when dry, cream to clay-buff pore surface when dry, and cylindrical to oblong-ellipsoid basidiospores (4–5 × 1.9–2.4 μm).

*Holotype* — **MALAYSIA**. Kuala Lumpur, Forest Eco-Park, on fallen angiosperm trunk, 14 April 2018, *Dai 18559* (BJFC 026848).

*Etymology* — “*submeliae*” (Lat.): refers to the new species resembling *Fomitopsis meliae* in morphology.

*Fruiting body* — Basidiomata annual, effused-reflexed with several small imbricate pilei protruding from a large resupinate part, inseparable from the substrate, corky, without odor or taste when fresh, corky to fragile and light in weight when dry. Single pileus up to 2 cm, 3.8 cm wide, and 6 mm thick at base; resupinate part up to 12 cm long, 4.5 cm wide, and 2.4 mm thick at center. Pileal surface cream when fresh, becoming buff to buff yellow when dry, rough, azonate; margin cream to buff, acute, incurved. Pore surface cream to pinkish buff when fresh, becoming cream to clay-buff when dry; pores round to angular, 4–7 per mm; dissepiments thick, entire to slightly lacerate. Context cream to buff, corky, up to 4 mm thick. Tubes concolorous with pore surface, corky to fragile, up to 2 mm long. Tissues unchanged in KOH.

*Hyphal structure* — Hyphal system dimitic; generative hyphae bearing clamp connections; skeletal hyphae IKI–, CB–.

*Context* — Generative hyphae infrequent, hyaline, thin-walled, rarely branched, 2–3.5 μm in diam; skeletal hyphae dominant, hyaline to pale yellowish, thick-walled with a wide to narrow lumen, rarely branched, more or less straight, interwoven, 2.6–6.4 μm in diam.

*Tubes* — Generative hyphae infrequent, hyaline, thin-walled, occasionally branched, 1.8–3 μm in diam; skeletal hyphae dominant, hyaline, thick-walled with a wide to narrow lumen, rarely branched, more or less straight, interwoven, 2–5 μm in diam. Cystidia absent; cystidioles occasionally present, fusoid, hyaline, thin-walled, 14.5–18 × 3.2–5 μm. Basidia clavate, bearing four sterigmata and a basal clamp connection, 15.8–21.5 × 4.8–6.5 μm; basidioles dominant, similar to basidia but smaller.

*Spores* — Basidiospores cylindrical to oblong-ellipsoid, hyaline, thin-walled, smooth, IKI–, CB–, (3.8–)4–5(–5.2) × 1.9–2.4(–2.6) μm, L = 4.49 μm, W = 2.11 μm, Q = 1.92–2.42 (*n* = 90/3).

*Type of rot* — Brown rot.

*Notes* — Five samples of *Fomitopsis submeliae* from China, Malaysia, and Vietnam formed a highly supported subgroup (99% ML, 100% MP, 1.00 BPP), and then grouped with *F. cana*, *F. meliae* and *F. srilankensis* (Figure 1). Morphologically, *F. cana* differs by having effused-reflexed and grayish basidiomata, pale mouse-gray to dark gray pileal surface, a trimitic hyphal system and larger basidiospores (5–6.2 × 2.1–3 μm; Li et al., 2013); *F. meliae* differs in having pileate basidiomata with an ochraceous pore surface and larger basidiospores (6–8 × 2.5–3 μm; Gilbertson, 1981; Núñez and Ryvarden, 2001); *F. srilankensis* differs in its pale mouse-gray to honey-yellow pileal surface, buff to cinnamon-buff pore surface when dry and larger basidiospores (5.5–6.6 × 1.9–2.5 μm). *Fomitopsis subtropica* B.K. Cui & Hai J. Li also distributes in China, Malaysia, and Vietnam, but *F. subtropica* differs from *F. submeliae* by having smaller pores (6–9 per mm) and smaller basidiospores (3.2–4 × 1.8–2.1 μm), a trimitic hyphal system (Li et al., 2013); in addition, it is distant from *F. submeliae* in the phylogenetic analyses (Figure 1).

Additional specimens (paratypes) examined — **CHINA**. Hainan Province, Baoting County, Tropical Garden, on fallen angiosperm trunk, May 27, 2008, *Dai 9719* (IFP 007971); Qiongzhong County, Limushan Forest Park, on fallen angiosperm trunk, 24 May 2008, *Dai 9544* (BJFC 007830); on rotten angiosperm wood, May 24, 2008, *Dai 9535* (BJFC 010339); *Dai 9543* (BJFC 010338); on angiosperm wood, May 24, 2008, *Dai 9525* (BJFC 007818). **VIETNAM**. Hochiminh, Botanic Garden, on angiosperm stump, October 12, 2017, *Dai 18324* (BJFC 025847).

**FIGURE 6:**
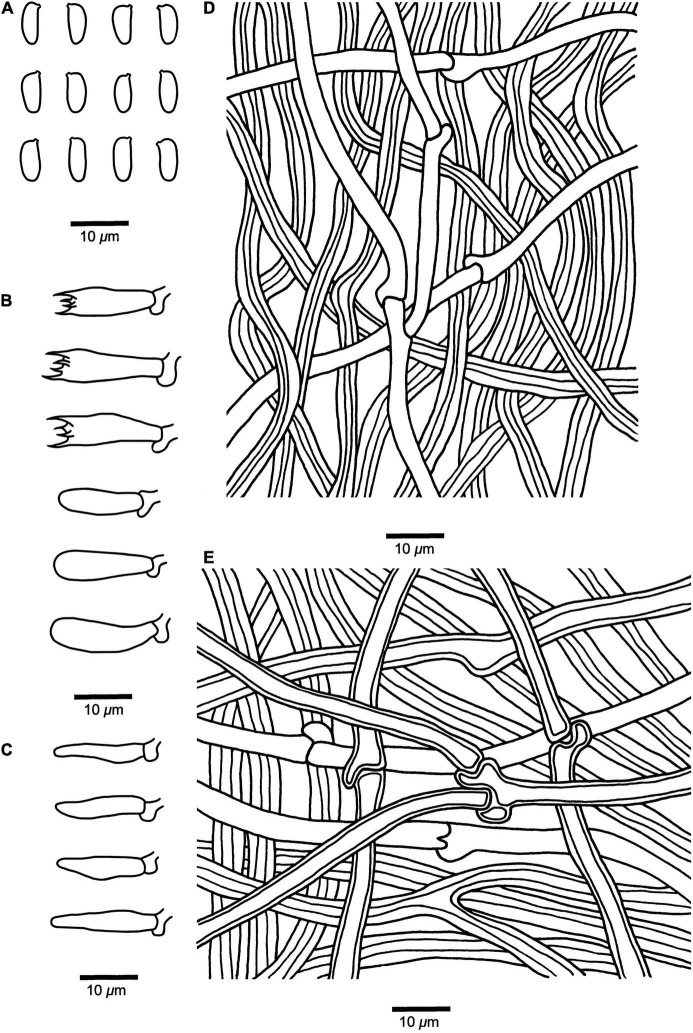
Microscopic structures of *Fomitopsis yimengensis* (Holotype, *Cui 5027*). **(A)** Basidiospores. **(B)** Basidia and basidioles. **(C)** Cystidioles. **(D)** Hyphae from trama. **(E)** Hyphae from context. Drawings by: Shun Liu.

***Fomitopsis yimengensis*** B.K. Cui & Shun Liu, **sp. nov.** (Figures 2E,F, 6).

MycoBank: MB 842876.

*Diagnosis* — ***Fomitopsis yimengensis*** is characterized by its pileate, solitary or imbricate basidiomata with pinkish buff, clay-buff to grayish-brown pileal surface, cream to pale cinnamon pore surface, thin-walled to slightly thick-walled generative hyphae in context, cylindrical basidiospores (6–7.2 × 2–3 μm).

*Holotype* — **CHINA**. Shandong Province, Mengyin County, on stump of *Pinus* sp., July 28, 2007, *Cui 5027* (BJFC 003068).

*Etymology* — “*yimengensis*” (Lat.): refers to the species distributed in Yimeng Mountains.

*Basidiomata* — Basidiomata annual, pileate, solitary or imbricate, without odor or taste when fresh, becoming hard corky and light in weight when dry. Pilei semicircular to flabelliform, projecting up to 2.8 cm long, 5.7 cm wide, and 1.7 cm thick at base. Pileal surface pinkish buff, clay-buff to grayish-brown, glabrous or with irregular warts, azonate; margin obtuse, cream to honey-yellow. Pore surface cream to pale cinnamon; pores round, 4–6 per mm; dissepiments thick, entire. Context cream to buff-yellow, corky, up to 1.2 cm thick. Tubes concolorous with pore surface, hard corky, up to 5 mm long. Tissues unchanged in KOH.

*Hyphal structure* — Hyphal system dimitic; generative hyphae bearing clamp connections; skeletal hyphae IKI–, CB–.

*Context* — Generative hyphae infrequent, hyaline, thin-walled to slightly thick-walled, occasionally branched, 2.2–4 μm in diam; skeletal hyphae dominant, yellowish brown to cinnamon brown, thick-walled with a wide to narrow lumen, occasionally branched, straight, 2.2–6.2 μm in diam.

*Tubes* — Generative hyphae infrequent, hyaline, thin-walled, occasionally branched, 1.9–3.3 μm in diam; skeletal hyphae dominant, hyaline to pale yellowish, thick-walled with a wide to narrow lumen, rarely branched, more or less straight, 1.9–4 μm in diam. Cystidia absent, but fusoid cystidioles occasionally present, hyaline, thin-walled, 13.8–18 × 2.8–4.2 μm. Basidia clavate, with a basal clamp connection and four sterigmata, 15.5–18 × 4.9–6.5 μm; basidioles dominant, similar to basidia but smaller.

*Spores* — Basidiospores cylindrical, hyaline, thin-walled, smooth, IKI–, CB–, 6–7.2 × 2–3(–3.1) μm, L = 6.64 μm, W = 2.71 μm, Q = 2.13–2.78 (*n* = 90/3).

*Type of rot* — Brown rot.

*Notes* — Three samples of *F. yimengensis* were successfully sequenced and formed a well-supported lineage (93% ML, 97% MP, 1.00 BPP), and then grouped with *F. caribensis* B.K. Cui & Shun Liu and *F. palustris* (Berk. & M.A. Curtis) Gilb. & Ryvarden (Figure 1). Morphologically, *F. caribensis* differs by having cream to pinkish buff pore surface when dry, round to angular and smaller pores (6–9 per mm), growth on angiosperm trees and distribution in the Caribbean regions (Liu et al., 2019); *F. palustris* differs in having malodorous fresh fruiting bodies, larger pores (2–4 per mm) and basidia (24–28 × 6–7 μm), and growth on angiosperm trees (Núñez and Ryvarden, 2001). *Fomitopsis yimengensis* and *F. bondartsevae* (Spirin) A.M.S. Soares & Gibertoni have similar basidiospores, but *F. bondartsevae* differs from *F. yimengensis* by having effused reflexed to pileate basidiomata, larger pores (2–3 per mm), a trimitic hyphal system and growth on *Tilia cordata* (Spirin, 2002). *Fomitopsis iberica* Melo & Ryvarden also grows on *Pinus* sp., but it differs from *F. yimengensis* by having larger pores (3–4 per mm), a trimitic hyphal system, larger cystidioles (20–27 × 4–5–5 μm), and has a distribution in Europe (Melo and Ryvarden, 1989).

Additional specimens (paratypes) examined — **CHINA**. Shandong Province, Mengyin County, Dongchangming, on stump of *Pinus* sp., July 28, 2007, *Cui 5031* (BJFC 003072); Mengyin County, Mengshan Forest Park, on fallen trunk of *Pinus* sp., August 6, 2007, *Cui 5111* (BJFC 003152).

## Discussion

In our current phylogenetic analyses, 30 species of *Fomitopsis* grouped together and formed a highly supported lineage (100% ML, 100% MP, 1.00 BPP; Figure 1). *Fomitopsis bondartsevae*, *F. caribensis*, *F. durescens*, *F. ginkgonis* B.K. Cui & Shun Liu, *F. hemitephra* (Berk.) G. Cunn., *F. iberica*, *F. nivosa*, *F. ostreiformis*, *F. palustris* and the two new species from China, viz., *F. resupinata*, *F. yimengensis* grouped together with high support (100% ML, 100% MP, 1.00 BPP; Figure 1); *F. cana*, *F. meliae* and the two new species, viz., *F. srilankensis*, *F. submeliae* formed a highly supported group (100% ML, 100% MP, 1.00 BPP; Figure 1); *F. roseoalba* A.M.S. Soares, Ryvarden & Gibertoni and *F. subtropica* formed a highly supported group (100% ML, 100% MP, 1.00 BPP; Figure 1); 10 species of the *F. pinicola* complex grouped together and formed a well-supported lineage (100% ML, 100% MP, 1.00 BPP) and related to *F. betulina* (Figure 1); *F. bambusae*, *F. eucalypticola* B.K. Cui & Shun Liu formed separate lineages, respectively (Figure 1). In addition, the current phylogenetic analyses also showed that *Fomitopsis* and other related brown-rot fungal genera clustered together within the antrodia clade, which are consistent with previous studies (Ortiz-Santana et al., 2013; Han et al., 2016; Liu et al., 2019, 2021a; Zhou et al., 2021).

*Fomitopsis* is a genus with important ecological functions and economic values. Since the establishment of the *Fomitopsis*, many new species and combinations had been described or proposed, and some *Fomitopsis* species have been removed to other genera. The taxonomic concept of *Fomitopsis* has been a subject of debate for a long time. Some species which previously belong to *Fomitopsis* are suggested to be excluded from the genus, such as, *F. concava* (Cooke) G. Cunn. (Cunningham, 1950), *F. maire* (G. Cunn.) P. K. Buchanan & Ryvarden (Buchanan and Ryvarden, 1988), and *F. zuluensis* (Wakef.) Ryvarden (Ryvarden, 1972). Although molecular data are not available for these species, their thick-walled basidiospores are quite different from the typical features of *Fomitopsis*. *Fomitopsis sanmingensis* is treated as a synonym of *F. pseudopetchii* (Lloyd) Ryvarden (Ryvarden, 1972). Although some species lack molecular data, the morphological descriptions are consistent with the *Fomitopsis* and remain in *Fomitopsis* according to previous studies, viz., *F. epileucina* (Pilát) Ryvarden & Gilb. (Ryvarden and Gilbertson, 1993), *F. minuta* Aime & Ryvarden (Ryvarden, 1972), *F. pseudopetchii* (Lloyd) Ryvarden (Ryvarden, 1972), *F. scortea* (Corner) T. Hatt. (Hattori, 2003), *F. singularis* (Corner) T. Hatt. (Hattori, 2003) and *F. subvinosa* (Corner) T. Hatt. & Sotome (Hattori and Sotome, 2013).

*Pilatoporus* Kotl. & Pouzar was established by Kotlába and Pouzar (1990) and typified by P. palustris (Berk. & M.A. Curtis) Kotl. & Pouzar based on the presence of pseudoskeletal hyphae with conspicuous clamp connections. Zmitrovich (2018) transferred *Fomitopsis cana*, *F. durescens*, *F. hemitephra*, *F. ostreiformis* and *F. subtropica* to *Pilatoporus*. However, there are no significant differences that can be found between *Pilatoporus* and *Fomitopsis* in morphology, and they grouped together in phylogeny (Figure 1). Thus, *Pilatoporus* is not supported as an independent genus and is considered as a synonym of *Fomitopsis* as previous studies show (Kim et al., 2005, 2007; Han et al., 2016).

**FIGURE 7:**
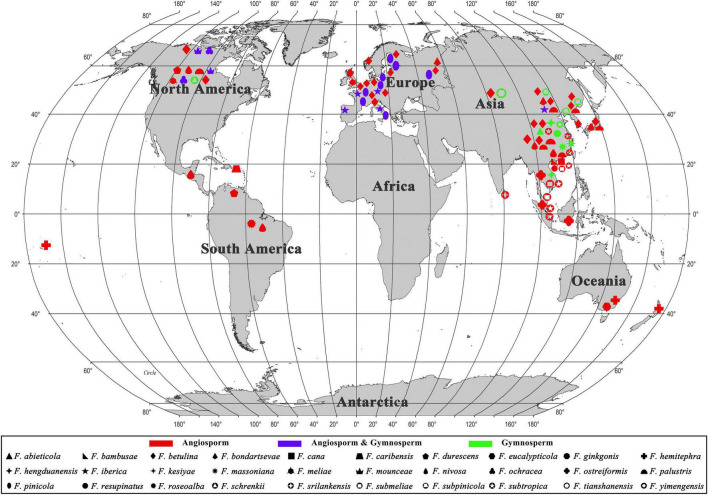
The geographical locations of the *Fomitopsis* species distributed in the world.

**FIGURE 8:**
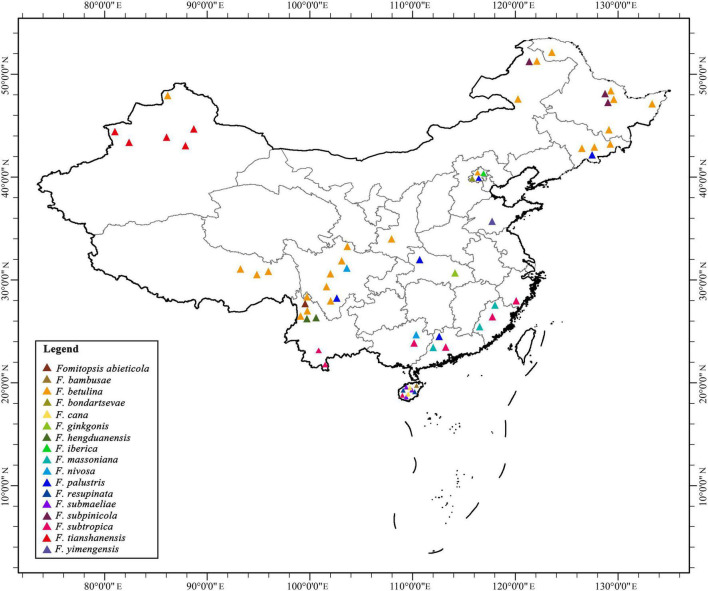
The geographical locations of the *Fomitopsis* species distributed in China.

During the investigations of *Fomitopsis*, the information of distribution areas and host trees were also obtained (Table 2), and the geographical locations of the *Fomitopsis* species distributed in the world and in China are indicated on the map, respectively (Figures 7, 8). The species of *Fomitopsis* have a wide range of distribution (distributed in Asia, Europe, North America, Oceania, South America; Table 2) and host type (grows on many different gymnosperm and angiosperm trees; Table 2). With regard to the geographical distribution, we found that 20 species of *Fomitopsis* are distributed in Asia, five in Europe, 10 in North America, three in South America and two in Oceania (Figure 7 and Table 2). Among the 20 species of *Fomitopsis* distributed in Asia, 17 are distributed in China, and 10 species are endemic to China (Figure 8 and Table 2). When analyzing the host type of the species of *Fomitopsis*, we found that all the species of *F. pinicola* complex can grow on gymnosperm trees, however, of the remaining species, only *F. ginkgonis*, *F. iberica* and *F. yimengensis* can grow on gymnosperm trees (Figure 1 and Table 2). Furthermore, some species of *Fomitopsis* have limited distribution areas and host specialization. In East Asia, *F. abieticola* is distributed in southwestern China and grows on *Abies* sp. (Liu et al., 2021a); *F. bambusae* is distributed in Hainan Province of China and grows on bamboo (Zhou et al., 2021); *F. cana* is distributed in Hainan Province of China and grows on *Delonix* sp. or other angiosperm wood (Li et al., 2013); *F. ginkgonis* is distributed in subtropical areas of Hubei Province of China and grows on *Ginkgo* sp. (Liu et al., 2019); *F. hengduanensis* is distributed in high altitude areas of the Hengduan Mountains of southwestern China and grows mostly on *Picea* sp. and other gymnosperm wood (Liu et al., 2021a); *F. kesiyae* is distributed in tropical areas of Vietnam and grows only on *Pinus kesiya* (Liu et al., 2021a); *F. massoniana* is distributed in subtropical areas of southeastern China and grows mainly on *Pinus massoniana* (Liu et al., 2021a); *F. resupinata* is distributed in Yunnan Province of China and grows on angiosperm wood; *F. srilankensis* is distributed in Sri Lanka and grows on angiosperm wood; *F. subpinicola* was found in northeastern China and grows mainly on *Pinus koraiensis* and occasionally on other gymnosperm or angiosperm wood (Liu et al., 2021a); *F. tianshanensis* is distributed in Tianshan Mountains of northwestern China and only grows on *Picea schrenkiana* (Liu et al., 2021a); *F. yimengensis* is distributed in Shandong Province of China and grows on *Pinus* sp. In North America, *F. caribensis* is distributed in the Caribbean regions and grows on angiosperm wood (Liu et al., 2019). In Oceania, *Fomitopsis eucalypticola* is distributed in Australia and grows on *Eucalyptus* sp. (Liu et al., 2019).

**TABLE 2:** The main ecological habits of *Fomitopsis* with an emphasis on distribution areas, host trees, and fruiting body types.

Species	Type locality	Distribution in the world	Distribution in China	Geographical elements	Host	Fruiting body types	References
*Fomitopsis abieticola*	China	Asia (China)	Yunnan (plateau humid climate)	Endemic to China	Gymnosperm (*Abies*)	Pileate	Liu et al., 2021a
*Fomitopsis bambusae*	China	Asia (China)	Hainan (tropical monsoon climate)	Endemic to China	Angiosperm (bamboo)	Resupinate to effused-reflexed or pileate	Zhou et al., 2021
*Fomitopsis betulina*	Norway	Asia (China, Japan, Korea), Europe (Austria, Belgium, Czech Republic, Finland, Germany, Italy, Lithuania, Norway, Russia, Switzerland, United Kingdom), North America (Canada, United States)	Beijing, Heilongjiang, Inner Mongolia, Jilin, Shaanxi, Sichuan, Xizang, Xinjiang, Yunnan (temperate to subtropical)	Cosmopolitan	Angiosperm (*Betula*)	Pileate	Ryvarden and Melo, 2014; present study
*Fomitopsis bondartsevae*	Russia	Asia (China), Europe (Russia)	Beijing (temperate continental monsoon climate)	East Asia-Europe	Angiosperm (*Prunus*, *Tilia*)	Pileate to effused-reflexed	Soares et al., 2017
*Fomitopsis cana*	China	Asia (China)	Hainan (tropical monsoon climate)	Endemic to China	Angiosperm (*Delonix*)	Resupinate to effused-reflexed or pileate	Li et al., 2013
*Fomitopsis caribensis*	Puerto Rico	North America (Puerto Rico)		North America	Angiosperm (undetermined)	Pileate	Liu et al., 2019
*Fomitopsis durescens*	United States	North America (United States), South America (Venezuela)		North America-South America	Angiosperm (*Fagus*)	Pileate	Gilbertson and Ryvarden, 1986
*Fomitopsis eucalypticola*	Australia	Oceania (Australia)		Oceania	Angiosperm (*Eucalyptus*)	Pileate to effused-reflexed	Liu et al., 2019
*Fomitopsis ginkgonis*	China	Asia (China)	Hubei (subtropical)	Endemic to China	Gymnosperm (*Ginkgo*)	Pileate	Liu et al., 2019
*Fomitopsis hemitephra*	New Zealand	Oceania (Australia, New Zealand, Samoa)		Oceania	Angiosperm (*Nothofagus*)	Pileate	Cunningham, 1965
*Fomitopsis hengduanensis*	China	Asia (China)	Yunnan (temperate to plateau continental climate)	Endemic to China	Gymnosperm (*Picea*)	Pileate	Liu et al., 2021a
*Fomitopsis iberica*	Portugal	Asia (China), Europe (Austria, France, Italy, Portugal)	Beijing (temperate continental monsoon climate)	Europe	Angiosperm (*Betula*, *Broussonetia*, *Prunus*), Gymnosperm (*Pinus*)	Pileate	Melo and Ryvarden, 1989; present study
*Fomitopsis kesiyae*	Vietnam	Asia (Vietnam)		Southeast Asia	Gymnosperm (*Pinus*)	Pileate	Liu et al., 2021a
*Fomitopsis massoniana*	China	Asia (China)	Fujian, Guandong (subtropical)	Endemic to China	Gymnosperm (*Pinus*)	Effused-reflexed to pileate	Liu et al., 2021a
*Fomitopsis meliae*	United States	Europe (United Kingdom), North America (United States)		Europe-North America	Angiosperm (*Prunuspersica*)	Pileate	Gilbertson, 1981
*Fomitopsis mounceae*	Canada	North America (Canada, United States)		North America	Angiosperm (*Betula*, *Populus*), Gymnosperm (*Abies*, *Picea*, *Tsuga*)	Pileate	Haight et al., 2019
*Fomitopsis nivosa*	Brazil	Asia (China, Japan), South America (Brazil), North America (Guatemala, United States)	Guangxi, Sichuan (alpine plateau to subtropical)	Cosmopolitan	Angiosperm (*Betula*, *Cinnamomum*, *Plum*, *Populus*, *Prunus*)	Pileate	Gilbertson and Ryvarden, 1986
*Fomitopsis ochracea*	Canada	North America (Canada, United States)		North America	Angiosperm (*Betula*, *Populus*), Gymnosperm (*Abies*, *Picea*, *Tsuga*)	Pileate	Stokland and Ryvarden, 2008; Haight et al., 2019
*Fomitopsis ostreiformis*	Philippines	Asia (Indonesia, Malaysia, Philippines, Thailand)		Southeast Asia	Angiosperm (*Cocos*)	Effused-reflexed to pileate	De, 1981; Hattori, 2003; Present study
*Fomitopsis palustris*	United States	Asia (China, Japan), North America (United States)	Beijing, Hubei, Guangdong, Jilin, Sichuan (temperate to subtropical)	East Asia-North America	Angiosperm (*Amygdalus*, *Ligustrum*, *Mangifera*, *Prunus*, *Tilia*)	Effused-reflexed to pileate	Corner, 1989; Hattori, 2003; present study
*Fomitopsis pinicola*	Sweden	Europe (Belgium, Czech Republic, Estonia, Finland, France, Italy, Poland, Russia, Sweden)		Europe	Angiosperm (undetermined), Gymnosperm (*Picea*, *Pinus*)	Pileate	Ryvarden and Melo, 2014; Haight et al., 2019; Present study
***Fomitopsis resupinata***	**China**	**Asia (China)**	**Hainan (tropical monsoon climate)**	**Endemic to China**	**Angiosperm (undetermined)**	**Resupinate**	**Present study**
*Fomitopsis roseoalba*	Brazil	North America (United States), South America (Venezuela)		South America	Angiosperm (undetermined)	Pileate, resupinate to effused-reflexed	Tibpromma et al., 2017
*Fomitopsis schrenkii*	United States	North America (United States)		North America	Angiosperm (undetermined), Gymnosperm (*Abies*, *Picea*, *Pinus*, *Pseudotsuga*)	Pileate	Haight et al., 2019
***Fomitopsis srilankensis***	**Sri Lanka**	**Asia (Sri Lanka)**		**South Asia**	**Angiosperm (undetermined)**	**Resupinate to effused-reflexed or pileate**	**Present study**
***Fomitopsis submeliae***	**China**	**Asia (China, Malaysia, Vietnam)**	**Hainan (tropical monsoon climate)**	**East Asia**	**Angiosperm (undetermined)**	**Resupinate to effused-reflexed or pileate**	**Present study**
*Fomitopsis subpinicola*	China	Asia (China)	Heilongjiang, Inner Mongolia, Jilin (boreal to temperate)	Endemic to China	Gymnosperm (*Pinus*)	Pileate	Liu et al., 2021a
*Fomitopsis subtropica*	China	Asia (China, Malaysia, Singapore, Vietnam)	Fujian, Guangdong, Guangxi, Hainan, Yunnan, Zhejiang (subtropical)	East Asia	Angiosperm (*Castanopsis*)	Resupinate to effused-reflexed or pileate	Li et al., 2013; present study
*Fomitopsis tianshanensis*	China	Asia (China)	Xinjiang (alpine plateau to continental climate)	Endemic to China	Gymnosperm (*Picea*)	Effused-reflexed to pileate	Liu et al., 2021a
***Fomitopsis yimengensis***	**China**	**Asia (China)**	**Shandong (temperate)**	**Endemic to China**	**Gymnosperm (***Pinus*)	**Pileate**	**Present study**

*New species are shown in bold.*

Fruiting body is one of the most significant morphological structures of fungi, which can protect developing reproductive organs and promote spore diffusion (Nagy et al., 2017). Previous studies have shown that the evolution of fruiting body types of higher taxonomic level (at or above the order level) in Basidiomycota have a trend from resupinate to pileate-stipitate (Hibbett and Binder, 2002; Hibbett, 2004; Nagy et al., 2017; Varga et al., 2019), however, few studies have explored the evolution of fruiting body types of specific families or genera. According to our observation of the fruiting body types of the species of *Fomitopsis*, we found that the species of *Fomitopsis* mainly with pileate or effused-reflexed basidiomata, and only *F. resupinata* produces completely resupinate basidiomata in the genus (Figure 1 and Table 2). The fruiting body types of *Fomitopsis* are similar to those of some genera of Fomitopsidaceae, such as *Buglossoporus* Kotl. & Pouzar, *Daedalea* Pers. and *Rhodofomes* (Han et al., 2016). We may draw a preliminary hypothesis that the ancestral of the *Fomitopsis* originated in Eurasia, with a pileate basidiomata and growth on gymnosperm trees. The current research cannot accurately reveal the ecological, morphological, and biogeographical evolution of *Fomitopsis*, which needs further study.

## Data Availability Statement

The datasets presented in this study can be found in online repositories. The names of the repository/repositories and accession number(s) can be found below: https://www.ncbi.nlm.nih.gov/genbank/, OL621842, OL621843, OL621844, OL621845, OL621846, OL621847, OL621848, OL621849, OL621850, OL621851, OL621852, OL621231, OL621232, OL621233, OL621234, OL621235, OL621236, OL621237, OL621238, OL621239, OL621240, OL621241, OL621745, OL621746, OL621747, OL621748, OL621749, OL621750, OL621751, OL621752, OL621839, OL621840, OL621841, OL621768, OL621769, OL621770, OL621771, OL621772, OL621773, OL621774, OL621775, OL621776, OL621777, OL621778, OL588960, OL588961, OL588962, OL588963, OL588964, OL588965, OL588966, OL588967, OL588968, OL588971, OL588972, OL588973, OL588974, OL588975, OL588976, OL588977, OL588978, OL588979, OL588980, and OL588981.

## Author Contributions

B-KC designed the experiment. SL, D-MW, and B-KC prepared the samples and drafted the manuscript. SL, C-GS, T-MX, and XJ conducted the molecular experiments and analyzed the data. All authors contributed to the article and approved the submitted version.

## Conflict of Interest

The authors declare that the research was conducted in the absence of any commercial or financial relationships that could be construed as a potential conflict of interest.

## Publisher’s Note

All claims expressed in this article are solely those of the authors and do not necessarily represent those of their affiliated organizations, or those of the publisher, the editors and the reviewers. Any product that may be evaluated in this article, or claim that may be made by its manufacturer, is not guaranteed or endorsed by the publisher.

## Footnotes

1
http://www.treebase.org

2
http://tree.bio.ed.ac.uk/software/figtree/

## Abbreviations

BI: Bayesian inference
BJFC: Herbarium of the Institute of Microbiology Beijing Forestry University
BGI: Beijing Genomics Institute
BPP: Bayesian posterior probabilities
BT: Bootstrap
CB: cotton blue
CB–: acyanophilous
GTR + I + G: general time reversible + proportion invariant + gamma
IKI: Melzer’s reagent
IKI–: neither amyloid nor dextrinoid
ILD: incongruence length difference test
ITS: internal transcribed spacer
KOH: 5% potassium hydroxide
L: mean spore length (arithmetic average of all spores)
ML: maximum likelihood
MP: maximum parsimony
MPT: most parsimonious tree
mtSSU: mitochondrial small subunit rRNA
n (a/b): number of spores (a) measured from given number (b) of specimens
nLSU: nuclear large subunit rDNA
nSSU: nuclear small subunit rRNA
Q: variation in the L/W ratios between the specimens studied
RPB2: DNA-directed RNA polymerase II subunit 2
TL: tree length
W: mean spore width (arithmetic average of all spores)
CI: consistency index
RI: retention index
RC: rescaled consistency index
HI: homoplasy index
TEF: translation elongation factor 1 −α.
